# Rapid Detection of *Mycoplasma bovis*, *Staphylococcus aureus* and *Streptococcus agalactiae* in Cattle Bulk Tank Milk in Cyprus and Relations with Somatic Cell Counts

**DOI:** 10.3390/pathogens10070841

**Published:** 2021-07-04

**Authors:** Maria Liapi, George Botsaris, Costas Arsenoglou, Nikolas Markantonis, Christodoulos Michael, Antonis Antoniou, Christodoulos Pipis

**Affiliations:** 1 Department of Agricultural Sciences, Biotechnology and Food Science, Cyprus University of Technology, Limassol 3036, Cyprus; mliapi@vs.moa.gov.cy (M.L.); nicolas.markantonis@cut.ac.cy (N.M.); ce.michael@edu.cut.ac.cy (C.M.); 2 Cyprus Veterinary Services, 1417 Athalassas Av, Nicosia 1417, Cyprus; carsenoglou@vs.moa.gov.cy (C.A.); hpipis@vs.moa.gov.cy (C.P.); 3 Cyprus Institute of Neurology and Genetics, Nicosia 1683, Cyprus; a.antoniou@nipd.com

**Keywords:** *Bovine mastitis*, *Mycoplasma bovis*, *Staphylococcus aureus*, *Streptococcus agalactiae*, somatic cells

## Abstract

One hundred and seventy-seven (177) bulk tank milk samples were analyzed with a commercially available real-time polymerase chain reaction kit and 11 (6.21%), 41 (23.16%), and 58 (32.77%) tested positive for *Mycoplasma bovis*, *Staphylococcus aureus,* and *Streptococcus agalactiae*, respectively. Statistical analysis revealed a significant relationship between the presence of *S. aureus* and *S. agalactiae*. Enumeration of somatic cells was performed in the same samples by flow cytometry. The somatic cell counts were found higher in *S. aureus* and *S. agalactiae* positive samples. No association was found between *M. bovis* presence and somatic cells counts. Low internal assay control Ct values were found to be related with high somatic cell counts. Noticeably, this is the first report for the presence of *M. bovis* in Cyprus. Therefore, its presence was confirmed by bulk tank milk culture, conventional PCR, and next generation sequencing. Furthermore, *M. bovis* was typed with multilocus sequencing typing and was allocated to sequence type 29 (ST 29). Real-time PCR in bulk tank milk samples is a useful tool to detect mammary infections, especially for neglected pathogens such as *M. bovis*.

## 1. Introduction

Mastitis is the most common disease of dairy cows, which results in economic losses to farmers and several animal welfare issues. Researchers and farmers have been making continuous efforts concerning mastitis detection, management, and prevention, since the beginning of modern dairy farming [1]. Mastitis is mainly caused by the bacterial species *Staphylococcus aureus* (*S. aureus*), *Streptococcus agalactiae* (*S. agalactiae*), *Mycoplasma* spp., *Escherichia coli*, *Klebsiella* spp., *Enterobacter aerogenes*, *Streptococcus uberis*, *Serratia* spp., *Pseudomonas* spp., *Proteus* spp. and other environmental Streptococci, and *Enterobacter* spp. [2,3,4]. *S. agalactiae* and *S. aureus* are considered to be the most significant contagious pathogens that can cause mastitis in cattle. Therefore, management control strategies have been developed for these pathogens. However, control still often depends on the use of antibiotics, which must be limited [1]. *Mycoplasma bovis* (*M. bovis*) is considered an emerging but overlooked cattle pathogen, which causes, in addition to mastitis, clinical disease with variable symptoms, including pneumonia, arthritis, genital disorders, keratoconjunctivitis, and otitis media [5]. Treatment of mastitis due to *M. bovis* with antibiotics is difficult and culling remains the most common recommendation [6].

Current research on gap analysis of *M. bovis* disease has highlighted the need for surveys to estimate the presence of *M. bovis*, since most of the available information is emanating from passive surveillance data [6]. The development of molecular methods, and specifically the introduction of the more recent probe based real-time PCR methods in bulk tank milk (BTM), has improved the sensitivity of mastitis pathogens detection compared to culture, whilst demonstrating also a high specificity [7,8,9]. So far, real-time PCR has been applied for the detection of mastitis pathogens in BTM only in a limited number of studies [10,11,12].

The presence of *S. aureus* and *S. agalactiae* in milk has also been additionally associated with elevated somatic cell counts (SCC), which have been also considered a useful tool for mastitis control programs [1]. In contrast, concerning *M. bovis*, recent studies suggest that SCC in milk are not sensitive enough to be used as herd level surveillance tools for mycoplasma mastitis and as a single basis for *M. bovis* mastitis control programs in farms [13,14,15].

A recent meta-analysis concludes that based on the current methods applied for the detection and identification of bacterial species, molecular methods are more sensitive than the traditional culture methods and should be considered as a diagnostic tool in future studies [2]. The real-time PCR assay used in this study includes an internal assay control (IAC), based on a ruminant housekeeping gene, which serves as an extraction and amplification control. It is suggested by the manufacturer that a low Ct value of the IAC may indicate a high somatic cell count in the milk sample tested. In case of negative results for the three pathogens, this information may additionally suggest an infection with other mastitis causing pathogens. However, no field study has been performed to confirm or reject this suggestion.

In Cyprus, there is no previous study describing the current situation regarding the presence of mastitis causing pathogens in raw milk. Considering all of the above, the objectives of this study were: (1) to detect *S. aureus*, *S. agalactiae* and *M. bovis* in cattle BTM samples using real-time PCR and estimate their prevalence; (2) to investigate the correlations of their presence or absence; (3) to evaluate BTM SCC in relation to the real-time PCR results for these three pathogens; and (4) to investigate the correlation of the Ct values of the internal assay control (IAC) of the real-time PCR assay and the SCC.

## 2. Results

### 2.1. Real-Time PCR Results

#### 2.1.1. Prevalence of Pathogens

All detectable species by the real-time PCR kit were identified. *S. agalactiae* was the most common pathogen found in 58/177 BTM samples (32.77%; 95% CI: 25.91–40.21%), followed by *S. aureus* in 41/177 BTM samples (23.16%; 95% CI: 17.16–30.08%) and *M. bovis* in 11/177 BTM samples (6.21%; 95% CI: 3.14–10.85%). *S. agalactiae* was present in BTM samples from all five districts, *S. aureus* was not found in BTM samples from one district (Paphos) and *M. bovis* was not found in BTM samples from two districts, (Limassol and Paphos) (see Table 1).

#### 2.1.2. Correlation of Presence or Absence between Pathogens

The comparisons of the results between the three pathogens are presented in Table 2 and were tested for statistical significance using the chi-squared test. The chi-square test between *M. bovis* and the other two pathogens did not fulfill the minimum expected cell frequency assumption, because of the low number of positive samples and, therefore, a safe conclusion could not have been drawn from this. However, the presence of *S. aureus* was low to moderately correlated with the presence of *S. agalactiae* in the BTM samples tested (Pearson’s correlation coefficient = 0.444). Following this, binary logistic regression was applied, and the likelihood of a *S. agalactiae* positive BTM sample to be found also positive for *S. aureus* was 8.917 (OR 8.917; 95% CI: 4.055 to 19.608) times higher than the likelihood of a *S. agalactiae* negative BTM sample to be found also positive for *S. aureus*.

#### 2.1.3. Comparison of SCC with the Presence and Absence of Each Pathogen

The independent samples t-test was conducted to compare SCC counts with the presence or absence of each pathogen. There was significant difference in SCC between positive *S. aureus* BTM samples (mean = 5.75, SD = 0.26) and negative *S. aureus* BTM samples (mean = 5.54, SD = 0.28, *p* = 0.00). Overall, nine-point one percent (9.1%) of the variability in BTM SCC was explained by the *S. aureus* real-time PCR status (positive/negative) of the BTM sample (eta squared = 0.091).

Regarding *S. agalactiae*, a significant difference in SCC was observed between positive (mean = 5.75, SD = 0.25) and negative BTM samples (mean = 5.52, SD = 0.27, *p* = 0.00). More specifically, thirteen-point eight percent (13.8%) of the variability in BTM SCC was explained by the *S. agalactiae* real-time PCR status (positive/negative) of the BTM sample tested (eta squared 0.138). For *M. bovis*, no significant difference was observed between the SCC of *M. bovis* positive (M = 5.63, SD = 0.11) and negative samples (mean = 5.59, SD = 0.29, *p* = 0.634).

#### 2.1.4. Correlation between the Ct Values of the IAC and the SCC

Following the statistical analysis, IAC Ct values and SCC were found to be significantly related variables with a Pearson’s correlation coefficient of 0.549.

#### 2.1.5. *M. bovis* Isolation, Identification and MLST Typing

The isolation of *M. bovis* from the dairy herds in Cyprus was never previously reported. Thus, following the positive PCR results, samples from positive herds were cultured in solid and liquid media in an attempt to isolate and characterize the pathogen for the first time. Three samples developed colonies in the solid media with the characteristic fried-egg appearance of the class mollicutes. DNA was extracted from the liquid media and PCR was performed for *M. bovis* and *Mycoplasma* spp. Two out of the three isolates were confirmed to be *Mycoplasma* spp. and specifically *M. bovis*. The other isolate was negative to both *Mycoplasma* spp. and *M. bovis* PCR.

DNA was further sequenced and tested for species identification with CCmetagen and the *M. bovis* DNA was tested with pubMLST. The cultured sample that was negative for *Mycoplasma spp.* and *M. bovis* DNA, was identified as *Acholeplasma laidlawii*, while both *M. bovis* DNA were species confirmed and allocated to *M. bovis* ST29.

## 3. Discussion

Bacterial culture of milk samples on blood agar plates has for many years been the standard method for isolation of mastitis pathogens, despite the fact that some pathogens, such as *Mycoplasma* spp., are not detected because they require specific media and growth conditions [16]. The sensitivity of BTM culture for mastitis pathogens is generally considered low and multiple BTM sampling is recommended to estimate the true prevalence of mastitis-causing pathogens [17,18].

The more recently developed probe based real-time PCR assays for detection of pathogens in BTM, have improved the sensitivity of mastitis pathogens detection compared to culture with a high specificity and allow the simultaneous detection of fastidious organisms, such as *M. bovis* [8,9,19].

The current literature review reveals three different studies which applied probe based real-time PCR in BTM for mastitis pathogens. One study in Canada, which includes the detection of the three mastitis pathogens examined here (*M. bovis*, *S. aureus,* and *S. agalactiae*) and two other studies, in Denmark and in three provinces of China, report the detection of *S. aureus* and *S. agalactiae* [10,11,12]. Comparatively, the prevalence of *M. bovis* in BTM found in this study (6.25%) was higher than the one found in BTM in Canada (0.53%). Concerning *S. aureus*, the prevalence in BTM in Cyprus (23.16%) was found to be lower than those reported in Canada (46%), Denmark (91%), and China (50.1%). For *S. agalactiae*, the prevalence in BTM (32.77%) was much higher than those reported in Canada (0.26%) and Denmark (7%) and much lower than the one found in China (92.2%). A more recent study in Egypt reports a total prevalence of 35.9% for *S. aureus* in cattle and additionally highlights the need to further investigate this pathogen for antimicrobial resistance [20]. This study did not look at the antimicrobial resistance patterns of the three mastitis pathogens detected, as the primary objective was to detect and estimate the extent of the presence of the pathogens tested. In the future, antimicrobial resistance should be addressed as it is a major concern regarding the public health issues involved with the development of antimicrobial resistance in the animal population and the food chain in general. Dairy farming in Cyprus has grown significantly over recent years, with the reported population of 28825 lactating animals in 2015 rising to 38473 in 2020 [21].

The present study shows that mastitis prevention control programs should be considered in Cyprus, as the population of animals increases and farming becomes more intensive. The low prevalence of *S. agalactiae* in BTM in countries like Denmark and Canada most probably reflects the impact of application of *S. agalactiae* prevention programs or the generally good management practices that are adopted for mastitis in these countries [12,22]. Additionally, the levels of *S. aureus* and *S. agalactiae* in China could be attributed to the different farming practices adopted there, due to the weakness of applying unified and efficient good management practices and control programs [11]. Concerning the high prevalence of *S. aureus* in BTM, it should be noted that contamination of BTM samples with *S. aureus* can lead to false positive results because *S. aureus*, apart from the udder, may have an environmental source in contrast to *S. agalactiae,* which is an obligate mammary pathogen [23]. The results of this preliminary study in Cyprus demonstrate a lower prevalence of *S. aureus* compared to the other studies but a higher *S. agalactiae* prevalence. This finding should alert the dairy industry stakeholders towards introducing control programs in an attempt to reduce *S. agalactiae* infection in dairy herds.

Overall, the presence of *S. aureus* and *S. agalactiae* in BTM in Cyprus was an expected finding since their presence is a common finding in milk samples from clinical cases of mastitis (veterinary services-unreported data). In contrast, the presence of *M. bovis* in Cyprus was unknown until this study. Therefore, samples from positive herds were cultured to isolate the pathogen and verify its presence in Cyprus. PCR identification and next generation sequencing of the isolates were additionally performed to confirm the presence of this pathogen and type it with MLST.

A recent study comparing the performance of the two MLST schemes available for *M. bovis* concludes that the originally used pubMLST scheme offers higher discriminatory power to that provided by the Weybridge scheme [24]. According to the pubMLST database, the *M. bovis* ST type 29 was identified in this study, which has also been found in five isolates from Israel and one isolate from an animal being quarantined in Israel after being imported from Hungary [24]. However, the dominant *M. bovis* MLST type in Israel was found to be ST10 [25]. The *Acholeplasma laidlawii* isolate, which was also identified, is generally considered a non-pathogenic contaminant, occasionally found in cattle milk samples [26]. More research is needed, which will include more isolates to better describe the distribution of the *M. bovis* and other *Mycoplasma* spp. strains in Cyprus.

Following the analysis of the results, the likelihood of a *S. agalactiae* positive BTM sample to be found also positive for *S. aureus*, was 8.917 times higher than the likelihood of a *S. agalactiae* negative BTM sample to be found also positive for *S. aureus*. The correlation between these two pathogens could be explained from common epidemiological risk factors and confirms other published suggestions [27]. In the present study, no conclusion could be drawn for correlations of *M. bovis* with the other two pathogens, due to the low number of positive *M. bovis* BTM samples. However, previous research suggests that *Mycoplasma* spp. must have a distinct epidemiology and a different set of risk factors than the more traditional contagious pathogens *S. aureus* and *S. agalactiae* [27].

This study confirms the previously reported association of *S. aureus* and *S. agalactiae* real-time PCR detection with elevated SCC in BTM samples [12]. Concerning *M. bovis* and SCC, earlier studies suggested that infection with *M. bovis* can elevate SCC in individual and BTM samples [28]. Therefore, the generally recommended rule to control intramammary infections due to *M. bovis* is sampling of cows with high SCC in milk [29]. However, more recent studies in individual milk suggest that due to the finding that not all culture positive animals show higher SCC, if SCC are used as the single basis for a mastitis control program in a farm, they may lead to the omission of *M. bovis* positive animals. Consequently, concerning SCC in BTM, it is suggested that SCC are not sensitive enough to be used as a surveillance tool for mycoplasma mastitis detection at herd level [13,14,15]. In the current study, no statistically significant association was found between *M. bovis* presence and SCC in BTM samples despite the fact that SCC counts were higher in *M. bovis* positive BTM samples.

Finally, the present study confirms an association between the IAC Ct values of the real-time PCR assay and the somatic cell counts. This finding can be a useful indicator in case of negative results for the three mastitis pathogens. The low Ct values in these cases, suggest the need for further diagnostic investigation of other mastitis pathogens. Subclinical mastitis caused by pathogens, such as *S. aureus*, *S. agalactiae*, and especially *M. bovis*, has a worldwide animal welfare and a public health significance [2]. The results from this investigation suggest that there is an urgent need to reduce the prevalence of mastitis pathogens by ensuring scientific farm management practices, proper feeding techniques, and therapeutic interventions. These measures could result in enhanced profits in a fast-growing dairy sector and improves animal and human health.

## 4. Materials and Methods

### 4.1. Cattle Population

During the sampling period, Cyprus had a total of 238 dairy cattle herds, which corresponded to a population of 23,093 animals in lactation at the time of sampling. Herd size was ranging between 20 and 2974 cows. All of the bovine animals were registered on the official database of the Cyprus Veterinary Services (see Table 1).

### 4.2. Sampling of BTM

One hundred and seventy-seven (177) BTM samples were collected from 177 dairy cattle herds through the mandatory milk quality surveillance scheme. Sampling was performed between February 2018 and May 2018. The samples were collected by the BTM truck drivers using automated truck milk collection equipment, in 40 mL sterile containers with 0.133 mL azidiol and stored at 4 °C until they were transferred to the laboratory for enumeration of somatic cells. Samples were then frozen at −20 °C and thawed prior to further processing. The 177 sampled herds corresponded to a population of 18,744 animals in lactation at the time of sampling. Herd size ranged between 24 and 1773 cows.

### 4.3. Enumeration of Somatic Cells

Enumeration of somatic cells in samples was determined by flow cytometry, using the Fossomatic™ FC instrument (Foss, Hillerød, Denmark). SCC were measured in cells/mL.

### 4.4. DNA Extraction and Real-Time PCR

The DNA extraction and the real-time PCR were performed with DNeasy Mastitis Mini Kit, Qiagen and Bactotype Mastitis HP3 PCR Kit (Qiagen), according to the manufacturer’s instructions. The Ct- value cut-off was set to be <35 for positive result. The thermal cycler used was the iQ5 (Bio-Rad).

### 4.5. M. bovis Isolation

Isolation of *M. bovis* was performed on Mycoplasma Experience solid and liquid Media (RH2 9BY Reigate, Surrey, UK), as previously described [30]. Colonies were seen using a stereo microscope (Zeiss stemi SV 11).

### 4.6. PCR’s and Next Generation Sequencing

For the next generation sequencing, DNA was extracted from the liquid media using the QIAamp UCP Pathogen Mini Kit (Qiagen). The PCR for the *M. bovis* (urvc gene) and the *Mycoplasma* spp. genus specific PCR were performed as described in the literature [31,32]. Next generation sequencing was performed with the i-Seq (Illumina) sequencer using the Nextera DNA Flex Library Preparation (Illumina). Species identification was performed using the CCmetagen [33,34]. MLST was performed using pubMLST [24].

The genomes described in this manuscript have been deposited in the National Center for Biotechnology Information (NCBI) under the project accession number PRJNA673092 with accession numbers SRR12937688, SRR12937689 and SRR12937690.

### 4.7. Statistical Analysis

The statistical analysis was performed using the SPSS 23 software (SPSS Inc., Chicago, IL, USA). Confidence intervals were estimated with the exact method [35]. SCC values and IAC Ct values were normalized by log transformation.

## 5. Conclusions

The real-time PCR in BTM is a useful, practical tool to detect mastitis pathogens in BTM. This study estimated a higher prevalence of *S. agalactiae* than *S. aureus* in Cypriot BTM samples from dairy cattle farms, and revealed the presence of *M. bovis* on the island, a neglected mastitis pathogen. Elevated SCC were confirmed to be related with real-time PCR detection of *S. aureus* and *S. agalactiae*, however they seem to have low usefulness for *M. bovis* infection. When real-time PCR is used as a diagnostic tool, low IAC Ct values can indicate a high SCC and even if a sample is negative for the pathogens tested, the presence of other mastitis pathogens should be investigated. Next generation sequencing is an extremely useful tool for one step identification or MLST typing of isolates, especially for challenging organisms such as mycoplasmas and Acholeplasmas.

## Figures and Tables

**Table 1:** Prevalence of *M. bovis*, *S. aureus* and *S. agalactiae* in BTM samples in Cyprus.

						Real-Time PCR Results			
				*M. bovis*		*S. aureus*		*S. agalactiae*	
District	No. of Existing Farms	No. of BTM Samples Collected	% Sampling Coverage	No. of Positive BTM Samples	Positive % (95% CI)	Number of Positive BTM Samples	Positive % (95% CI)	No. of Positive BTM Samples	Positive % (95% CI)
Nicosia	93	63	67.74	3	4.76 (0.99–13.29)	11	17.46 (9.05–29.1)	17	26.98 (16.57–39.65)
Limassol	11	10	90.91	0	0	6	60 (26.24–87.84)	6	60 (8.29–28.52)
Larnaca	79	68	86.08	5	7.35 (2.43–16.33)	19	27.94 (17.73–40.15)	25	36.76 (25.39–49.33)
Famagusta	40	32	80	3	9.38 (1.98–25.02)	5	15.63 (5.28–32.79)	8	25 (11.46–43.4)
Paphos	5	4	80	0	0	0	0	2	50 (6.76–93.24)
**Totals**	**228**	**177**	**77.63**	**11**	**6.21** **(3.14–10.85)**	**41**	**23.16** **(17.16–30.08)**	**58**	**32.77** **(25.91–40.21)**

**Table 2:** Comparison of mastitis pathogen detection in BTM samples.

		*S. agalactiae*		*M. bovis*	
		Negative BTM Samples	Positive BTM Samples	Negative BTM Samples	Positive BTM Samples
***S. aureus***	Negative BTM samples	107	29	128	8
	Positive BTM samples	12	29	38	3
***M. bovis***	Negative BTM samples	110	56	-	-
	Positive BTM samples	9	2	-	-

## Data Availability

Not applicable.
